# The Ranking Probability Approach and Its Usage in Design and Analysis of Large-Scale Studies

**DOI:** 10.1371/journal.pone.0083079

**Published:** 2013-12-20

**Authors:** Chia-Ling Kuo, Dmitri Zaykin

**Affiliations:** 1 Center for Quantitative Medicine, University of Connecticut Health Center, Farmington, Connecticut, United States of America; 2 Biostatistics Branch, National Institute of Environmental Health Sciences, Research Triangle Park, North Carolina, United States of America; National Taiwan University, Taiwan

## Abstract

In experiments with many statistical tests there is need to balance type I and type II error rates while taking multiplicity into account. In the traditional approach, the nominal 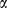-level such as 0.05 is adjusted by the number of tests, 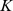, i.e., as 0.05/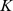. Assuming that some proportion of tests represent “true signals”, that is, originate from a scenario where the null hypothesis is false, power depends on the number of true signals and the respective distribution of effect sizes. One way to define power is for it to be the probability of making at least one correct rejection at the assumed 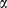-level. We advocate an alternative way of establishing how “well-powered” a study is. In our approach, useful for studies with multiple tests, the ranking probability 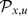 is controlled, defined as the probability of making at least 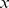 correct rejections while rejecting hypotheses with 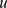 smallest P-values. The two approaches are statistically related. Probability that the smallest P-value is a true signal (i.e., 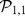) is equal to the power at the level 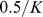, to an excellent approximation. Ranking probabilities are also related to the false discovery rate and to the Bayesian posterior probability of the null hypothesis. We study properties of our approach when the effect size distribution is replaced for convenience by a single “typical” value taken to be the mean of the underlying distribution. We conclude that its performance is often satisfactory under this simplification; however, substantial imprecision is to be expected when 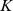 is very large and 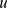 is small. Precision is largely restored when three values with the respective abundances are used instead of a single typical effect size value.

## Introduction

Development of novel statistical methods to accompany technological advances in biological sciences is essential in order to further scientific progress. Human genetics is a prime example of a field where data is currently being produced at an accelerating rate. The sheer volume of data and its complexity create new challenges for statistical science. How to design studies appropriately, and how not to drown in the data while extracting useful signals? Studies now routinely involve multiple testing of statistical hypotheses on a massive scale. Thus, both design and analysis of large-scale experiments must entail considerations of multiplicity.

**Figure 1:**
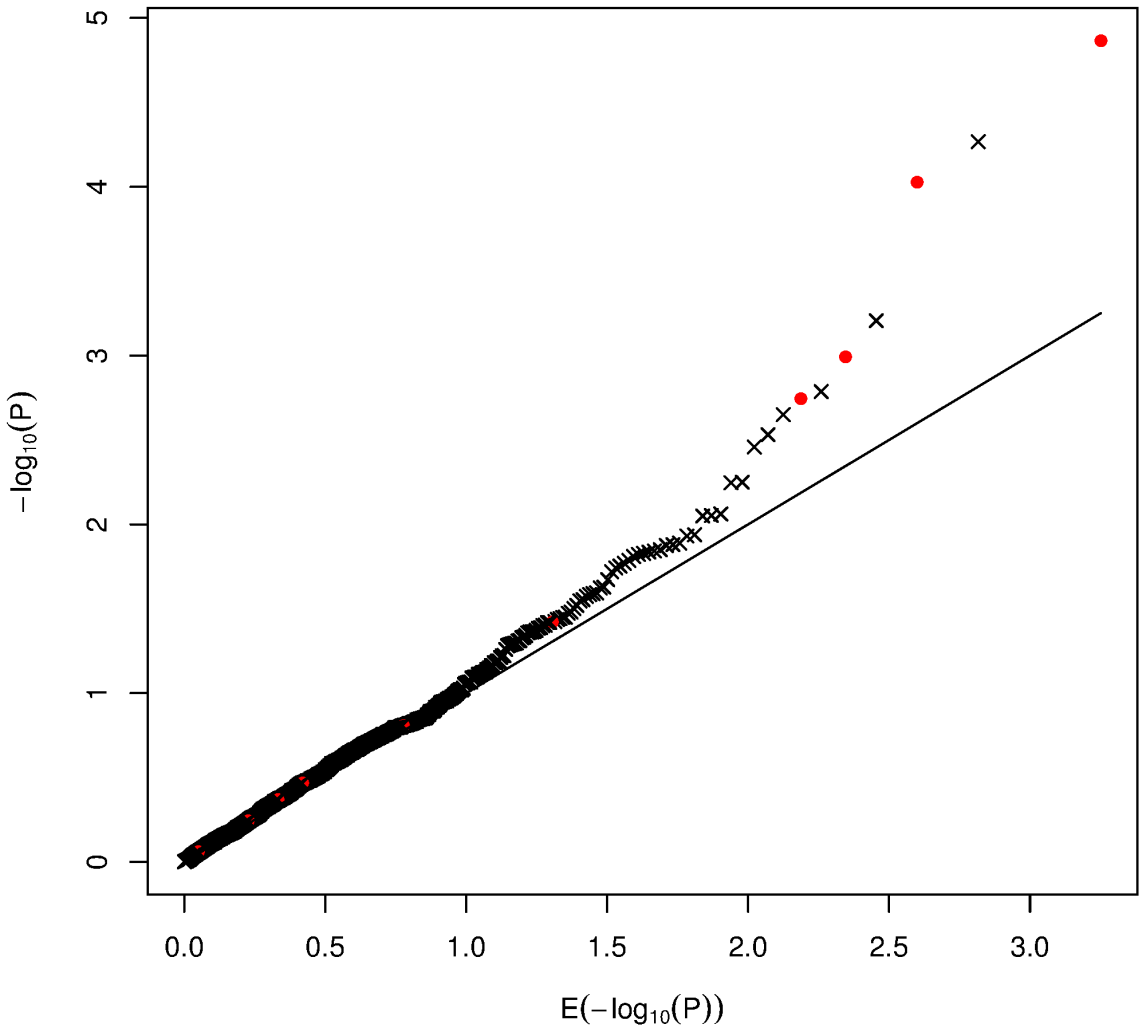
Simulated plot of −log_10_ (P-value). red circles– true signals; crosses– false signals.

Design of studies often involves sample size and power calculations. In exploratory studies where many statistical tests are anticipated, it is reasonable to assume that most test statistics and their respective P-values are generated under the condition where the null hypothesis holds. One would also assume that there are multiple real signals. In reality, effect sizes (e.g. odds ratios) for these true signals follow some distribution, although it might be tempting to assume, for simplicity, a single “typical” effect size. In multiple testing contexts, statistical power can be defined as the probability of correctly rejecting at least one (or at least 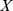) null hypotheses. Power defined this way increases with the total number of true signals. Thus, not only the total number of tests, but the number of truly associated loci and the distribution of effect sizes must be taken into account. Figure 1 is an illustrative example that shows a sample 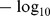
(P-value) plot for a simulation experiment with multiple tests. The graph shows ordered 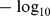 of 1000 P-values (Y axis) for a one degree of freedom chi-square statistic, plotted against what is expected under the null hypotheses (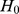), i.e. when P-values would follow the uniform (0,1) distribution. Ten out of 1000 P-values, denoted by red circles, were simulated assuming the alternative hypothesis so that these P-values originated from statistics having a noncentral chi-square distribution. The largest value (4.9), that happened to be a true positive, is the only one significant at the level of 0.05/1000. Yet, among the first ten largest values, there are four true signals. These observations raise some important statistical questions:

1. What is the probability that the largest value on the graph is a true signal?
2. What is the expected number (or proportion) of true signals among, for example, the first ten top results?

We will seek a simple and practical way with a minimum number of assumptions to allow for such computations.

In the simulation experiment to construct Figure 1, ten noncentralities for the ten assumed true signals were sampled from the Gamma(1/2, 15) distribution. Given such information, the questions above can be answered exactly, with the help of simple formulas that will be detailed later. Specifically, probability that the minimum P-value (top red dot) is a true signal is equal to 0.93, and the expected proportion of true signals among the first ten top results is 0.35 (the actual proportion observed in this particular experiment is 4/10). Because the noncentrality is the multiple of sample size (Equation 4), one can also calculate a sample size needed for a specified proportion of true signals to aggregate among a speciffied number of top hits.

For the next motivating example, we considered a genome-wide association study (GWAS) with 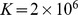 association trend tests among which there were 75 true signals with the allele frequency 0.15 and the relative risk 1.15. We assumed 3000 cases, 3000 controls, which resulted in the noncentrality 7.8 that governed the distribution of P-values for true signals. Under these assumptions,

- Power to detect any particular associated variant at the 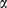-level of 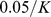 is only 0.003.
- Power to detect at least one of the 75 true signals is 0.19, at the 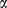-level of 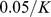.

More revealing are calculations based on the ranks of true signals:

- Probability that the best hit (i.e. the smallest among 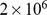 P-values) is a true signal is 0.5.
- There is 0.95 probability that at least one true signal will be encountered among the 11 smallest P-values.
- Expected proportion of true signals among these 11 smallest P-values is 0.23, i.e. there are about 2–3 true signals expected among the 11 smallest P-values.

**Figure 2:**
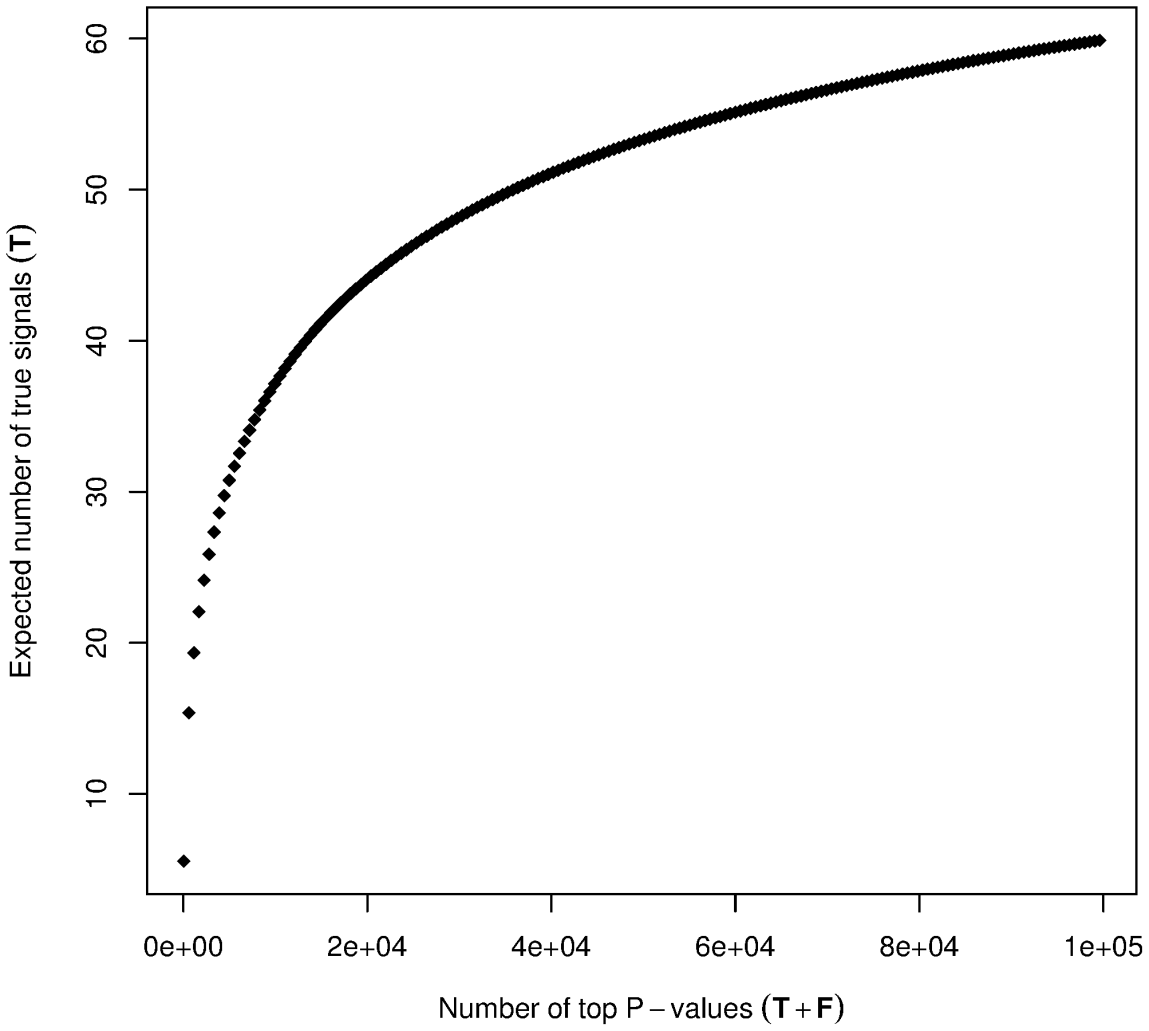
Number of true signals in a set of top P-values. The number of true signals (*T*) expected to be encountered among a specific number of the smallest P-values.

Table 1 shows an expanded list for the number of true signals (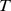) expected to be encountered among a specific number of the smallest P-values (that would contain both true and false signals, 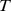
+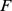). These numbers are also shown in Figure 2. The number of real discoveries grows sharply at first; however, a large number of P-values would be required to “cover” a good portion of the 75 true signals. At the same time, the proportion of true signals among top P-values, 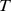/(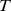
+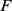), quickly drops. There is a good chance to find some true signals at the top of the list of best results, but only a few of the 75 can be found. If we suppose a confirmatory study of top hits, then following up a large number of results would again incur a substantial multiple testing penalty.

**Table 1:** Number of true signals in a set of top P-values.

*u*	*E*(*T*)	*u*	*E*(*T*)
50	5.5	7200	34.1
600	15.4	7750	34.8
1150	19.3	8300	35.4
1700	22.1	8850	36.0
2250	24.1	9400	36.6
2800	25.9	9950	37.1
3350	27.3	10500	37.7
3900	28.6	11050	38.2
4450	29.7	11600	38.6
5000	30.8	12150	39.1
5550	31.7	12700	39.5
6100	32.6	13250	40.0
6650	33.3	13800	40.4

*E*(*T*): expected number of true signals among top *u* P-values.

The total number of tests: 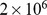; the total number of true signals: 75.

All of the calculations for the presented examples are derived from the ranking probability, i.e. the probability that at least 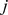 true signals would rank among the top 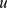 hits, 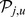 [1]. As these examples illustrate, ranking probability is a concept that allows characterization of how well-powered a study is in a way that is usefully different from the regular power calculation. The ranking approach depends on the same assumed parameters as the statistical power approach, yet it provides additional information about a study. Ranking probabilities are related to the false discovery rate (FDR), in particular, the proportion of true signals among a specified number of top P-values can be expressed via the average of ranking probabilities. We provided approximations to 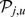 and showed good performance of these approximations under local dependence of P-values, such as dependence due to linkage disequilibrium in GWAS [1]. Here, we specifically address the issue of accuracy in estimating the expected proportion of true signals among top hits of a study. A major difficulty with applications of the approach is its reliance on a parametric distribution used to characterize the effect size distribution [1]. Not only it is difficult to estimate parameters of such a distribution reliably, but the effect of misspecification of that distribution has been unclear. A distribution can be specified erroneously in a multitude of possible ways: not only the parameter values, but the distribution itself might be incorrect. Here we addressed the issue of misspecification by taking a perspective that very little might be guessed well about the effect size distribution, perhaps only its mean or the abundance of a few effect size values, and evaluated performance of the ranking approach under these conditions. With only one or a few specific effect size values, the approach simplifies considerably and can be more readily implemented and applied for design of genetic studies.

## Materials and Methods

For ease of exposition, we assume one-degree of freedom chi-squared statistics, commonly found in genetic association studies. P-values are random variables, with the cumulative distribution function (CDF) given by
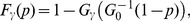
(1)where 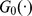 and 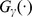 denote the CDF of the test statistic under the null and the alternative hypothesis and 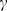 is the noncentrality parameter. When 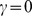, we get the uniform distribution, 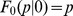. P-value density for a fixed 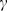 can be obtained by differentiating 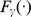:
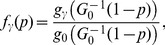
(2)where 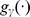 is the density that corresponds to the cumulative distribution 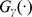. The inverse of Equation (1) can be used conveniently to generate random P-values under the alternative hypothesis, i.e. assuming a certain noncentrality, as
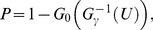
(3)where 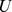 is a random number from the Uniform(0,1) distribution [2]. The noncentrality parameter can be calculated assuming an effect size, such as odds ratio, and a particular statistic. For example, the usual 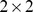 chi-square statistic for testing log odds ratio (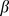) being equal to zero has the noncentrality that is approximately
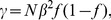
(4)where 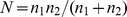, i.e. 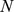 is one half of the harmonic mean of the counts for the table row totals (they are assumed to represent two binomial samples) and 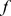 is the frequency, pooled across the rows. Recently, Lee and Wray reported simple formulas for computing the noncentrality given various experimental designs [3].

### Ranking probability

Equation (1) gives the P-value distribution for a fixed value of the noncentrality, 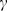. Different signals have distinct associated noncentrality values. When different tests have approximately the same sample size, as in GWAS, noncentralities can be thought to arise from some distribution, 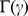.

For continuously distributed effect sizes, Gamma(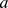,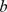) distribution can be utilized. This distribution provides flexible shapes, and for its shape parameter values 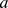 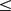
1, the distribution is L-shaped, which corresponds to a biologically realistic assumption that there are many small effects, but a few that are large. The ranking probability calculation makes use of the marginal P-value distribution, averaged over all possible noncentrality values,
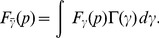
(5)Similarly, assuming that noncentralities for true signals arise from some distribution, 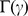, the marginal density of P-values for true associations is
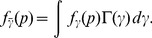
(6)


Effect size distribution may be specified in a tabulated fashion and statistical methods begin to emerge for estimating the effect size distribution, discretized into 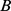 bins (e.g. [4]). Each bin is defined by the average effect size, 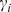, and the relative abundance of signals that fall into that bin, 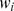. The discrete version of the marginal distribution can thus be defined as follows:
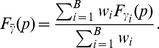
(7)


When true signals are independent, the CDF of the 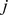-th ordered true signal P-value is
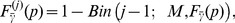
(8)where Bin denotes the binomial CDF evaluated at 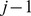 successes in 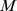 trials (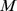 is the number of true signals) with the success probability 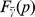. We previously derived a precise approximation for the ranking probability [1]. Specifically, ranking probability, as the chance that at least 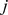 true signals will rank among top 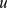 smallest P-values is
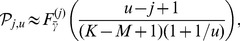
(9)(where 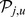 = 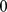 for 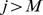). We note that although the ranking probability depends on the total number of true and false signals, as well as on the effect size distribution, only 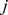 and 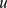 are the varying indices in a given study. Thus, we omit dependence on other parameters to make the notation succinct. The approximation given by Equation (9) improves as both the rank 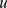 and the total number of tests 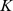 increase, but the error for 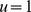 is only around 1–3% at 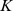 as small as 10 (data not shown). Even though our focus is on large-scale testing, the ranking approach is valid when 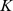 is small and provides an excellent approximation when 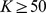 and tests are independent. If there is considerable local correlation between P-values, as found in large-scale association studies, the term 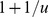 can be dropped for increased precision. The resulting formula still provides a good approximation, unless the correlation is extreme and 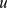 is close to one. In a study with 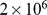 tests that behaved as only 6.4% of that number due to extreme correlation (as judged by the distribution of the minimum P-value), the approximation was adequate at 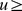
15 [1].

The main question that we address here is how the ranking approach is affected by the precision in specification of the effect size distribution 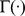 that is utilized by equations for the marginal P-value distribution (Equations 5, 6). Considering noncentralities (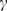
's) as random, we can rewrite Equation (5) as the expectation,
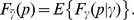
Using the first order Taylor approximation,
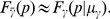
(10)where 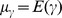. This is also Equation (7) with 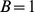. Approximations utilizing the mean introduce imprecision, compared to the approaches based on the distribution, but the degree of inaccuracy due to these approximations has been unclear.

### Relation between ranking probability, power, and the expected P-value

If we replace 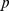 by a significance threshold 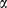, Equation (1) will define power at the 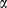 level for a particular signal with the noncentrality 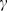. With multiple (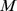) independent true signals, power to detect at least one of them is
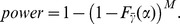

Equation (9) reveals a relation of probability that the smallest P-value is a true signal to power to detect a particular true signal. If 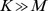, so that 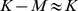,
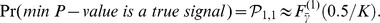
Thus, probability that a true signal ranks first when results of a multiple testing experiment are sorted by P-value is the average power to detect at least one signal at the level ten times higher than the conventional, Bonferroni-adjusted level, 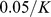.

The ranking approach can also be related to the “expected P-value” approach of Sackrowitz and Samuel-Cahn [5] who considered the probability that a single false signal P-value (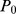) is smaller than a single true signal P-value (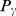). This probability can be written in terms of the expectation of the true signal P-values:
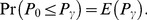
(11)In the context of multiple testing considered in our ranking approach, there are multiple false P-values. Let us denote the 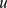-th smallest P-value (without regard whether that is a true or false signal) by 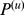. 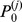 and 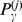 will denote the 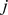-th smallest P-value among false and true signals, respectively. With this notation,
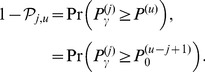
(12)Considering a particular true signal and setting 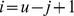,
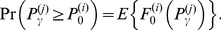
(13)

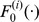
 is the CDF of the 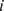-th ordered P-value under the null hypothesis, which for continuous and independent test statistics has the Beta(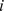, 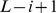) distribution, where 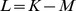 is the number of false signals. When there are only two tests, one of which is a false signal, Equation (12) is equal to Equation (11).

### Ranking false discovery rate (rFDR)

The ranking false discovery rate, rFDR(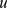) is defined as the proportion of null signals among the top 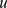 hits. A study-specific rFDR value is
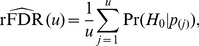
(14)where 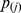 is the 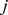-th smallest P-value, and 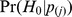 is the posterior probability of the null hypothesis given the 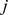-th smallest P-value. These posterior probabilities are unaffected by the fact of selection of the smallest P-values, that is, they do not depend on 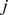, but only on the value 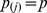:
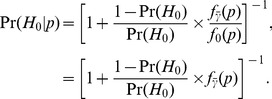
(15)The second equality assumes that under 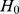, P-values follow the uniform (0,1) distribution. The rFDR can be related to the traditional FDR concept, introduced by Benjamini and Hochberg [6]. In their method, the proportion of false discoveries among discoveries is controlled by the expectation that is taken over multiple-testing experiments with or without rejections. Storey [7] confined attention to control of the FDR among only those experiments that contained rejections of 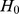 (“positive FDR”, pFDR). While the pFDR is defined for a fixed P-value threshold, the study-specific rFDR is defined for a fixed rank, 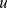. The expected rFDR(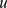) is defined as the average across many multiple-testing experiments:
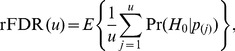
(16)

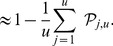
(17)
Equations (16), (17) show the relation between the posterior probability and the ranking probability. Approximation, rather than equality arises from the fact that Equation (17) assumes that the number true signals, 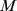, is fixed, while Equation (16) assumes that each P-value corresponds to a true signal with probability 1-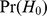, resulting in the *expected* number of true signals across multiple-testing experiments being equal to 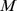. There is a slight difference in values provided by these equations when 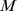 is close to one, hence the relation is approximate.

### Simulation experiments

We used simulation experiments to evaluate precision of rFDR estimates. These simulations were used to construct Tables 2, 3, 4. At each simulation, we sampled noncentrality parameters for a specific number of true signals from an L-shaped Gamma(shape = 1, scale = 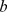) distribution. P-values for 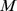 true signals were simulated using this sample of noncentralities using Equation (3), while P-values for the remaining 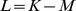 signals were sampled from the Uniform(0,1) distribution. Next, P-values were sorted, the 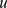 smallest P-values were taken and the proportion of true signals was recorded. 1-rFDR values were estimated via Equation (17). This describes a single simulation experiment. The 1-rFDR values were averaged across 1000 simulations to produce an estimate as given by Equation (16).

**Table 2:** 1-rFDR(*u*) by ranking probabilities for *u* = 100 top P-values using the distribution mean, or distribution of noncentralities for true effects where noncentralities of *M* = 100 true effects are distributed as Gamma(1,*b*).

*K*	*b*	true	rank_dist_	rank_bin_	rank*_μ_*
1e4	5	0.298	0.300	0.309	0.320
1e4	10	0.478	0.477	0.486	0.610
1e4	14	0.562	0.564	0.574	0.761
1e4	17	0.609	0.611	0.621	0.837
1e4	25	0.696	0.696	0.705	0.943
1e4	30	0.733	0.731	0.740	0.971
1e4	40	0.780	0.781	0.795	0.994
1e4	50	0.814	0.815	0.840	0.999
1e4	75	0.865	0.864	0.919	1.000
1e4	100	0.892	0.892	0.961	1.000
1e5	5	0.172	0.171	0.177	0.136
1e5	10	0.345	0.346	0.356	0.394
1e5	14	0.441	0.444	0.452	0.584
1e5	17	0.500	0.500	0.509	0.696
1e5	25	0.603	0.604	0.614	0.878
1e5	30	0.652	0.650	0.655	0.934
1e5	40	0.714	0.714	0.712	0.982
1e5	50	0.759	0.757	0.759	0.996
1e5	75	0.822	0.823	0.857	1.000
1e5	100	0.860	0.859	0.922	1.000
1e6	5	0.094	0.094	0.094	0.048
1e6	10	0.248	0.247	0.259	0.215
1e6	14	0.348	0.345	0.353	0.394
1e6	17	0.405	0.405	0.411	0.522
1e6	25	0.524	0.522	0.534	0.777
1e6	30	0.576	0.574	0.586	0.869
1e6	40	0.649	0.649	0.649	0.959
1e6	50	0.702	0.701	0.690	0.989
1e6	75	0.783	0.781	0.787	1.000
1e6	100	0.827	0.826	0.869	1.000

*K*: number of tests.

*b*: scale parameter of Gamma to model noncentralities for true effects (shape parameter is fixed at 1).

true: simulation-based 1-rFDR(100).

rank*_μ_*: 1-rFDR(100) by ranking probabilities using mean.

rank_bin_: 1-rFDR(100) by ranking probabilities using the 3-bins approximation.

rank_dist_: 1-rFDR(100) by ranking probabilities using the noncentrality distribution.

**Table 3:** 1-rFDR(*u*) by ranking probabilities for *u* = 200 top P-values using the distribution mean, or distribution of noncentralities for true effects where noncentralities of *M* = 100 true effects are distributed as Gamma(1,*b*).

*K*	*b*	true	rank_dist_	rank_bin_	rank*_μ_*
1e4	5	0.186	0.186	0.191	0.215
1e4	10	0.278	0.278	0.284	0.374
1e4	14	0.320	0.320	0.327	0.443
1e4	17	0.344	0.343	0.350	0.471
1e4	25	0.383	0.382	0.391	0.496
1e4	30	0.397	0.398	0.410	0.499
1e4	40	0.419	0.420	0.439	0.500
1e4	50	0.435	0.434	0.461	0.500
1e4	75	0.454	0.454	0.489	0.500
1e4	100	0.464	0.465	0.497	0.500
1e5	5	0.105	0.105	0.109	0.094
1e5	10	0.199	0.199	0.204	0.248
1e5	14	0.248	0.249	0.254	0.350
1e5	17	0.276	0.277	0.283	0.406
1e5	25	0.330	0.329	0.333	0.479
1e5	30	0.350	0.350	0.354	0.493
1e5	40	0.382	0.381	0.385	0.499
1e5	50	0.401	0.401	0.413	0.500
1e5	75	0.430	0.430	0.463	0.500
1e5	100	0.447	0.446	0.487	0.500
1e6	5	0.057	0.058	0.058	0.034
1e6	10	0.140	0.141	0.147	0.138
1e6	14	0.193	0.192	0.196	0.241
1e6	17	0.223	0.223	0.227	0.311
1e6	25	0.281	0.282	0.288	0.436
1e6	30	0.306	0.307	0.312	0.473
1e6	40	0.345	0.344	0.343	0.496
1e6	50	0.370	0.370	0.367	0.500
1e6	75	0.407	0.407	0.424	0.500
1e6	100	0.428	0.428	0.465	0.500

*K*: number of tests.

*b*: scale parameter of Gamma to model noncentralities for true effects (shape parameter is fixed at 1).

true: simulation-based 1-rFDR(200).

rank*_μ_*: 1-rFDR(200) by ranking probabilities using mean.

rank_bin_: 1-rFDR(200) by ranking probabilities using the 3-bins approximation.

rank_dist_: 1-rFDR(200) by ranking probabilities using the noncentrality distribution.

**Table 4:** 1-rFDR(*u*) by ranking probabilities for *u* = 10 top P-values using the distribution mean, or distribution of noncentralities for true effects where noncentralities of *M* = 100 true effects are distributed as Gamma(*a*,*b*).

*K*	*a*	*b*	true	rank_dist_	rank_bin_	rank*_μ_*
1e4	0.7	3	0.447	0.433	0.406	0.259
1e4	0.9	3	0.564	0.553	0.531	0.365
1e4	0.7	5	0.760	0.754	0.749	0.512
1e4	0.9	5	0.855	0.880	0.881	0.683
1e4	0.7	10	0.988	0.998	0.999	0.952
1e4	0.9	10	0.999	1.000	1.000	0.999
1e5	0.7	3	0.203	0.195	0.157	0.066
1e5	0.9	3	0.285	0.273	0.234	0.108
1e5	0.7	5	0.472	0.473	0.434	0.183
1e5	0.9	5	0.628	0.620	0.595	0.303
1e5	0.7	10	0.935	0.950	0.968	0.655
1e5	0.9	10	0.982	0.995	0.998	0.877
1e6	0.7	3	0.086	0.081	0.051	0.014
1e6	0.9	3	0.121	0.120	0.085	0.026
1e6	0.7	5	0.265	0.265	0.203	0.050
1e6	0.9	5	0.388	0.375	0.320	0.097
1e6	0.7	10	0.801	0.801	0.816	0.308
1e6	0.9	10	0.922	0.937	0.954	0.546

*K*: number of tests.

*a*: shape parameter of Gamma to model noncentralities for true effects.

*b*: scale parameter of Gamma to model noncentralities for true effects

true: simulation-based 1-rFDR(10).

rank*_μ_*: 1-rFDR(10) by ranking probabilities using mean.

rank_bin_: 1-rFDR(10) by ranking probabilities using the 3-bins approximation.

rank_dist_: 1-rFDR(10) by ranking probabilities using the noncentrality distribution.

In addition, for each simulation setting, the expected rFDR was estimated via the sum of ranking probabilities, by utilizing (1) the true (continuous) effect size distribution; (2) the distribution reduced to three bins (by utilizing Equation 7 with the bins defined as [0, 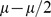], [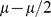, 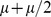], and [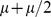, 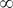]; and their relative sizes (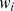) obtained from the integration on the true distribution); (3) the distribution reduced to its mean. The terms 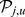 in Equation (17) were computed using the approximation given in Equation (9). Note that this computation does not utilize P-values and in that way it is analogous to a classical power computation.

In Figure 1, 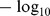 transformed ordered P-values were plotted against their expected values, computed under 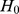. The −log transformation provides better visualization of deviations from the expected line, compared to the plots of non-transformed P-values. A reviewer raised the issue of a possible usage of 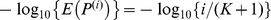 in place of 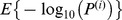. This appears to be a common practice due to simplicity of the resulting expression, however, bringing the expectation inside introduces a bias due to Jensen's inequality. This bias causes a tendency for the smallest null P-values to appear above the expected line. The correct expected values can be found as follows. As before, denote 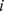-th smallest P-value by 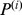. Under 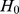, 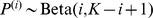 and 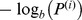 has the expectation
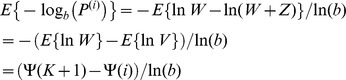
where 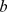 is the base of the logarithm, 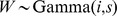, 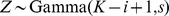, 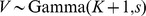, 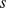 is an arbitrary scale parameter of the gamma distribution, and 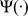 is the digamma function.

## Results

As shown in Materials and Methods, power calculations, ranking probabilities, Bayesian forms of FDR, and posterior probabilities of 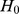 depend on the assumed distribution of effect sizes. The noncentrality can be regarded as a standardized effect size. Park et al. [4] explicitly defined the effect size to be 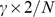, where 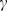 is defined by Equation (4). In the following, we will keep sample size 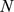 in the “effect size” definition, thus by “effect size” we will simply mean 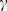.

In simulation experiments, we utilized the ranking false discovery rate, rFDR(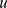), defined as the proportion of null signals among the top 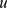 hits. We investigated imprecision in estimates of the proportion of true signals (1-rFDR) due to approximations by Equations (7, 10) via simulation experiments as detailed in Materials and Methods: Simulation Experiments. Tables 2, 3, 4 show the results for three simulation settings, where we considered 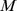 = 100 (the number of true signals) and 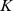 = 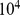, 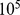, 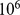 (total number of P-values). We conducted simulations assuming independence, however, as 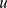 increases, ranking probabilities computed under the independence assumption become robust in the presence of even extreme correlation among false signals, which are assumed to represent majority of the tests.

In Tables 2, 3, we considered the number of top hits to be 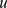 = 100 and 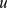 = 200, correspondingly; and in Table 4, 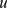 was set to 10. Noncentralities were assumed to arise from an L-shaped Gamma(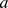, 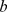) distribution. In Table 2, the shape was set to 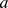 = 1 and the scale was varied. In Table 4, both the shape (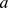) and the scale (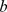) were varied. Smaller shape and scale values result in smaller mean values of the noncentralities (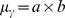), thus, in lower true values of 1-rFDR.

As expected, 1-rFDR computed by the method that utilizes the actual continuous effect size distribution (via Equation 5) is precise in all scenarios. The three bin approximation performed similarly to the integration method and consistently gave estimates close to the true 1-rFDR values. The error introduced by the simplified analysis, where instead of the integration of the noncentrality distribution, its mean value 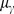 is utilized, gives acceptable results for relatively large values of top hits (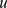 = 100 and 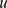 = 200). For 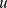 = 10, there appears to be considerable inaccuracy, especially when 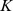 becomes as large as 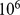.

**Figure 3:**
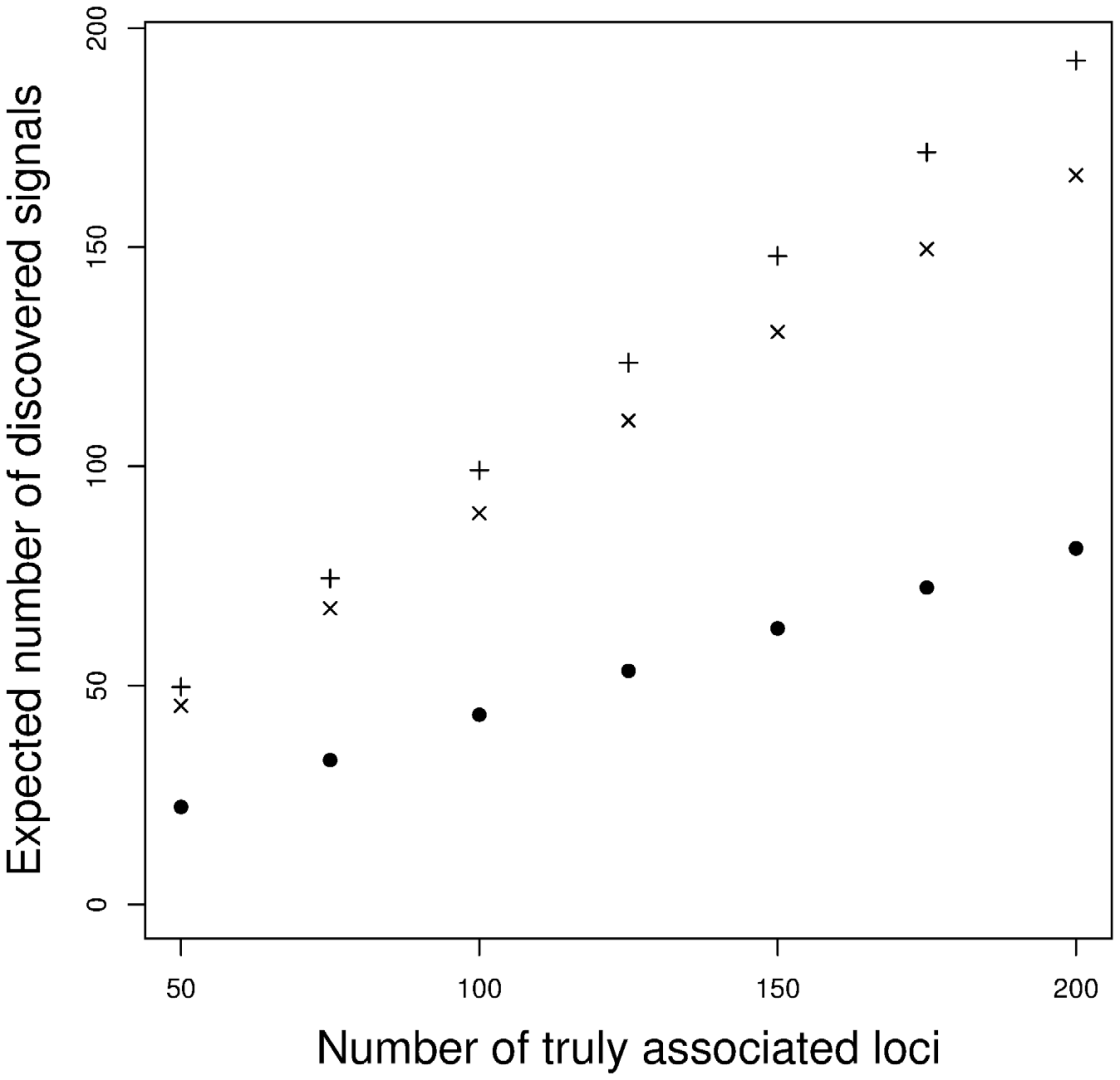
Expected number of true discoveries among the first 200 top results. •: 5000 cases and 5000 controls. ×: 10000 cases and 10000 controls. +: 15000 cases and 15000 controls.

Figure 3 shows the expected number of true signals among the first 200 top results, as a function of the number of truly associated signals. Assuming the single value approximation, we postulated the term 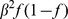 in Equation (4) to be equal to 0.0024, which could have resulted assuming that typical value of the odds ratio is 1.15 and the allele frequency 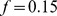. Further, we assumed 10^6^ tests and three different sample sizes. The smallest sample size (5000 cases and 5000 controls) allows to detect less than one half of the actual number of true signals. Doubling the sample size makes the number of discovered signals to rise considerably more sharply, as the number of true signals increases. The combined sample (15000 cases and 15000 controls) allows to detect nearly all true signals.

## Discussion

Classical counterparts to ranking and posterior probabilities are statistical power and P-values. Power is calculated for a fixed significance threshold while in our ranking approach it is the number of top hits that is fixed. “Fixed” in this context simply means not-random: it is possible to vary the number of top hits by incrementing 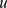 starting from one, until a predefined value of rFDR is reached, or until the ranking probability (e.g. 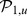) is at a needed value. Gail et al. provided an alternative approach for estimating the expected proportion of true signals among a fixed number of top hits of a study [8]. Our approach is conceptually the same as that of Gail et al., but differs in the implementation. Our approach utilizes P-values, and can accommodate variety of test statistics. It also provides additional flexibility in the way the effect size distribution is specified. In particular, the distribution is not limited to a normal, in fact, one can avoid usage of any parametric distribution by employing the tabulated effect size distribution (Equation 7). Our approach hinges on expressing the rFDR in terms of the ranking probability introduced in Zaykin and Zhivotovsky [2] and on the usage of approximations of Kuo and Zaykin [1]. Usage of the tabulated distribution (a typical effect size value, e.g., the distribution mean, is a special case) brings about additional computational simplicity, and we investigated performance of this approach as an approximation to the true continuous effect size distribution.

Ranking probabilities, rFDR, as well as standard power calculations when multiple tests are anticipated, all depend on the effect size distribution and the number of true signals. Specification of these parameters can be challenging. Here, we investigated whether the usage of the mean of the effect size distribution would provide acceptable precision. Wacholder et al. [9] proposed the “False Positive Report Probability” (FPRP), defined as the proportion of true signals among P-values that are smaller or equal to a fixed threshold 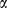. FPRP also utilizes a single “typical” effect size instead of using an assumed effect size distribution. This was criticized as an oversimplification [10], [11]. But usage of a single value taken to be the mean of the underlying prior distribution can be justified as the first order Taylor approximation to the method that takes into account the entire distribution (Equation 10). Our simulation experiments show that imprecision introduced by this simplification may be tolerable. Usage of the distribution mean instead of the entire distribution is much simpler computationally. From the practitioner's viewpoint, it is relatively easy to specify a “typical” effect size, that is the mean, rather than the distribution. In addition, it might be possible to estimate the mean of the effect size distribution more reliably than the distribution itself.

P-value distribution functions, 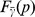 and 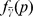, can also be expanded to the second order Taylor series, and in the case of the usual chi-square test statistic these approximations are easily computed:
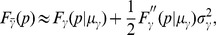


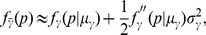
where 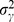 is the variance of 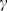. The expectation operator can be thought of as a summation of approximation values (weighted by the density of 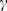 at each particular point) across the entire domain of 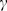. However, it is known that higher order expansions may work worse than lower order ones away from the point of expansion (in our case, 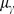). Our investigation into quality of these second order approximations did not reveal marked improvements over the first order approximations, especially at tiny P-values, where the second order ones sometimes performed worse (results not shown). Thus, in this paper, we focused on simple first order approximations. Satisfactory performance utilizing the mean of the effect size distribution suggests that our approach is robust against misspecification of the shape of the effect size distribution. However, there is considerable imprecision when 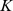 is very large and 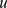 is small. This underlines importance of development of statistical methods for estimating parameters of the effect size distribution. While replacing a continuous effect size distribution by its mean value may result in a loss of precision, collapsing that distribution into three bins performed surprisingly well in all simulations. Correct specification of three typical values (low, medium, high) with the relative abundance of each appears sufficient for computing the proportion of true positives among top hits with high precision and can be recommended for practical use.

R programs to compute the expected number of true (truly associated) signals among top 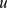 hits for a given sample size are available at https://sites.google.com/site/entrust2013/. The programs can also be used to choose a sample size such that a desired number of true signals would rank among the top 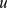 hits.
