# Correction: The Ranking Probability Approach and Its Usage in Design and Analysis of Large-Scale Studies

**DOI:** 10.1371/annotation/6bc48309-0280-4066-b937-e88debd1579c

**Published:** 2014-01-28

**Authors:** Chia-Ling Kuo, Dmitri Zaykin

This article was republished on January 27, 2014 due to a previous version being published. Please download this article again to view the correct version.

